# Exploring Rural Medicine Residency Programs: A Narrative Review

**DOI:** 10.7759/cureus.72238

**Published:** 2024-10-23

**Authors:** Sainamitha R Palnati, Chad M Jurado, Saajan H Bhakta

**Affiliations:** 1 Research, Kansas College of Osteopathic Medicine, Wichita, USA; 2 Languages and Literature, Eastern New Mexico University, Albuquerque, USA

**Keywords:** family medicine, family practice, graduate medical education, healthcare, kansas, medical practice, medical resident, physician shortage, residency program, rural physicians

## Abstract

The challenges of rural medicine are not unknown as access to healthcare is compounded by physician recruitment, specialty care, and resource availability. Rural populations across the US are in a constant struggle for proper medical care and preventive health. Despite research efforts to examine the practice of rural medicine, little has been shared about the role of medical education in overcoming the hurdles of rural medicine. A review of current literature is needed to better understand the current situation in rural medicine and how medical education can aid in addressing the challenges of rural medicine.

This review aimed to understand the practice of rural medicine, identify measures taken to address the physician shortage nationally in rural communities, and emphasize the role of rural medicine residency programs and training programs in addressing the current situation.

A review of the literature was carried out by searching several electronic databases, which included PubMed, ScienceDirect, Google Scholar, Directory of Open Access Journals (DOAJ), JSTOR, PsycINFO, ERIC Database (via EBSCOhost), and Academic Search Complete. The search was conducted from January 27, 2022, to February 28, 2022, using Boolean operations and the following terms: rural, family practice, healthcare, residency program, medical resident, graduate medical education, Kansas, and Midwest.

There were 23 relevant publications included in this review that explored the role of medical education, physician recruitment, and scope of practice in addressing the struggles of rural medicine. Rural medicine residency programs and targeted training play a crucial role in addressing the physician shortage in underserved areas. Future research should focus on thoroughly understanding rural communities’ interactions with the healthcare system and increasing support for medical professionals in rural areas.

## Introduction and background

Accessibility to healthcare for rural populations is a commonly known challenge that has long been tied to the ongoing maldistribution of physicians between rural and urban geographies [1]. In 2017, a Kansas state task force found 161 primary care health professional shortage areas in Kansas. Ninety-two of the state’s 105 counties are considered partially or wholly underserved. This review aims to open the dialogue for planning to address this gap.

At the national level, the challenge in the US has yet to be resolved despite ongoing efforts and research to better understand the complexities of the phenomenon since at least the 1980s. Efforts have taken various pathways, but one of the most prominent has been the creation of numerous rural training track (RTT) programs at medical schools around the country, which have sought to increase the number of rural-practicing physicians [2]. These programs have taken different approaches in terms of recruitment and training with varied results. Some programs have seen a fair amount of success with ongoing continuation, but others have, unfortunately, been shuttered [3]. The reasons for the ongoing success of established programs have been generally agreed upon within a broader context, but finite rationale has been opened to debate as shown in the following publications. Many of these articles highlight similar issues in terms of recruitment and retention, and they have found common ground in the emphasis on rural-background applicant recruitment, prolonged rural practice exposure, and financial incentives.

Although these factors are typically agreed upon, there remains room for further analysis as many of these articles tend to rely heavily on self-reported data, which brings about its own limitations due to challenges of bias. The purpose of this exploratory review was to better understand the current literature on this broad topic and work to address the rural practicing physician shortage in Kansas.

## Review

Methods

**Figure 1:**
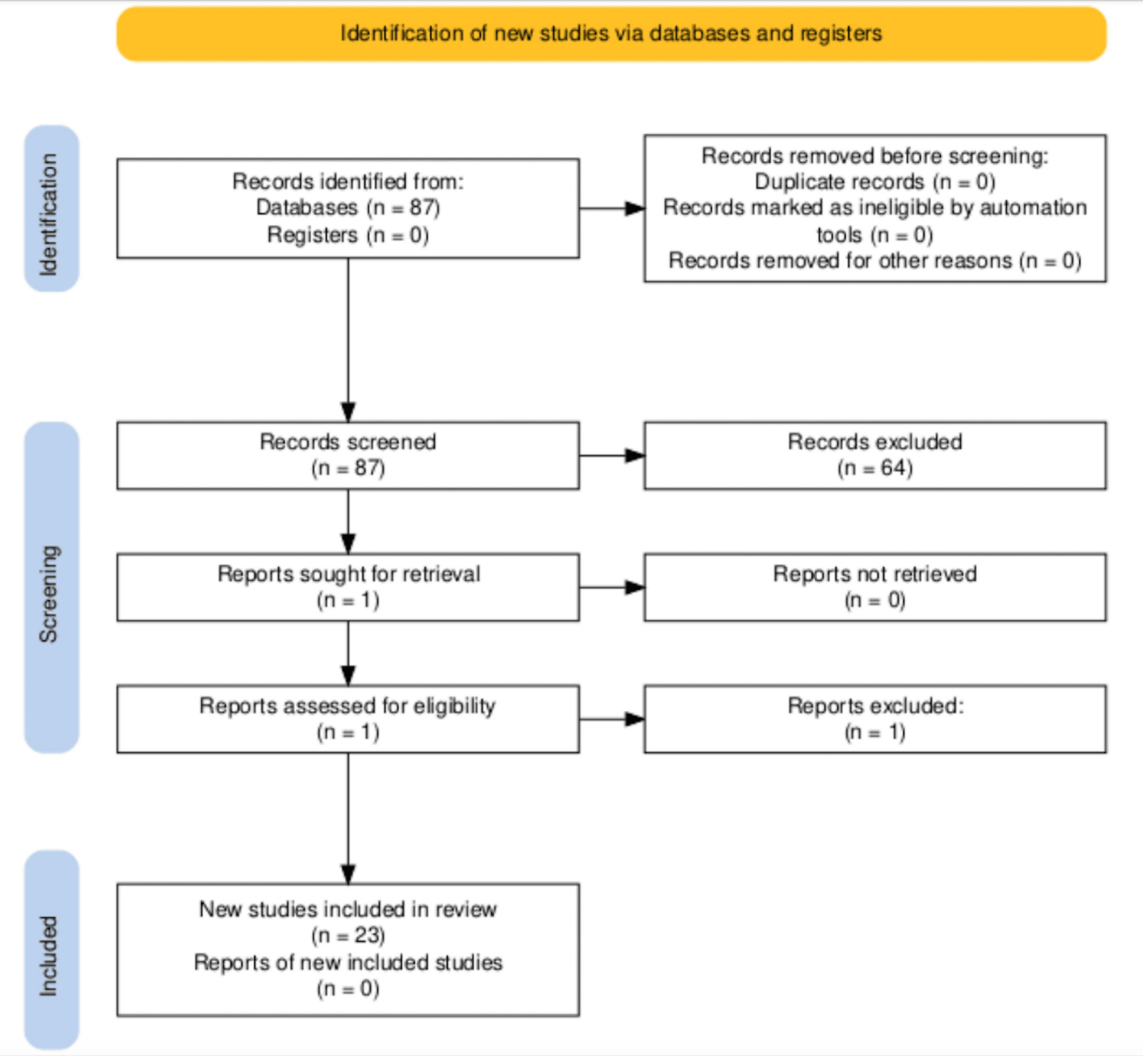
PRISMA flow diagram of the summarized search strategy n: number of studies, PRISMA: Preferred Reporting Items for Systematic Reviews and Meta-Analyses

The articles included in this review focus on strengths, limitations, lessons learned, and demographic attributes for developing rural residency and training programs. Electronic databases were searched from January 27, 2022, through February 28, 2022, for studies relating to rural medicine residency programs, factors related to graduates’ choices on where to practice, and important lessons for potential new rural programs. Databases included in the search were the following: PubMed, ScienceDirect, Google Scholar, Directory of Open Access Journals (DOAJ), JSTOR, PsycINFO, ERIC Database (via EBSCOhost), and Academic Search Complete. Keywords used for the search included: rural, family practice, healthcare, residency program, medical resident, graduate medical education, Kansas, and Midwest. Boolean operators were used as conjunctions to combine or exclude keywords to identify relevant abstracts. All relevant articles were included given the internal, exploratory, and scoping goal and nature of this review. The exclusion criteria consisted of articles not focused on medical education related to rural medicine, non-English publications, non-peer-reviewed studies, and articles with no full text available. Figure 1 depicts the search strategy used for this comprehensive review of 23 articles.

Literature review

A thorough review of the literature was completed to understand the current situation of rural medicine. This review highlights (i) rural vs. urban dichotomy of medical practice, (ii) rural medicine in medical education, (iii) scope of practice as rural physicians, and (iv) addressing physician storage in rural communities. The literature underscored the physician shortage in rural communities of Kansas.

Rural Versus Urban Dichotomy of Medical Practice

The rural-urban dichotomy is evident within the medical field. It affects medical education, medical practice and consultation, physician recruitment, and overall medical care for patients within the community.

Physician recruitment: In terms of tailoring rural residency programs to influence rural practice outcomes, Ogden et al. worked to quantify the effects of a rural upbringing and experience in rural areas during medical training on the likelihood of general practitioner (GP) recruitment and retention with a systematic review of existing literature. The review drew from literature that consisted of quantitative comparisons that focused on associations between rural factors and the location or duration of later clinical practice for GPs or family physicians [4]. This review did not include qualitative studies, case reports and series, abstracts, education articles, and opinion pieces. Qualitative studies were excluded due to their use of diverse methodologies and sampling strategies, as well as the potential for subjectivity in the interpretation of qualitative findings. Further exclusions were made if the desired GP or family physician-specific information could not be isolated or used proxy measures. In this review, the rural background was considered as having spent at least a year in a rural space or having graduated from a rural high school. Rural experience in the context of medical education was considered as completing a rotation or working in a rural setting for at least a year. Researchers used MEDLINE, EMBASE, Informit Health Collection, and ERIC databases in searching for prospective literature for inclusion. Initially, researchers identified 6,702 records for potential inclusion, with all but seven coming from electronic databases. Early screening removed 6,554 duplicates and irrelevant texts, leaving 155 full-text articles that were ultimately culled to 27 eligible studies for inclusion in the review. Twelve of the studies had cohort designs, four were case-controlled, and 11 were cross-sectional with the US, Canada, and Australia representing the set nearly evenly. These studies reported current rural practice location and rural retention as study outcomes [4]. Despite alignment on key components, there were substantial differences in the definitions given to rural location and retention in rural practice, as well as notable variation in the time spent in rural areas. The selected studies were generally well regarded except for three, which relied on self-reported information about rural experience and practice and did not adjust for confounding variables. Meta-analysis of the collected data sets indicated that GPs in rural communities were strongly associated with rural background and rural clinical experience [4].

Ogden et al. inferred that rural residency programs and medical schools can aid in increasing awareness and opportunities for rural exposure within their clinical rotations and training to yield an increase in rural practice [4]. These conclusions are not new by any means, but it does highlight the importance of rural exposure either before or during the completion of a medical training program in influencing rural practice outcomes. Although the review was well executed in terms of its framework, it is slightly hampered by the fact that the studies included were all observational and characterized by their heterogeneity. The variation in key terminology as well as the over-reliance on self-reported information made side-by-side comparisons more difficult to execute. Despite these complications, the review works well in summarizing the broader characteristics of research on this topic and guiding readers through the heterogeneous boundaries of investigation.

Acknowledging that rural training programs yield an increase in rural practicing physicians, Patterson et al. quantified the availability of rurally located training and rural content in residency programs that aimed to produce rural physicians in the rurally relevant specialties. While acknowledging the maldistribution of physicians in the US, they were primarily driven to conduct this study as a result of recent changes in the accreditation system managed by the Accreditation Council for Graduate Medical Education (ACGME). The decision by the council to move to a single allopathic and osteopathic accreditation system runs the risk of dismantling rural programs. This fear is brought about by the fact that these rural programs, which tend to produce more rural-practicing physicians than their urban counterparts, are more likely to lose accreditation due to lower program enrollment numbers. It is further exacerbated by the fact that osteopathic programs tend to have fewer residents than allopathic programs [5]. Researchers utilized FREIDA Online and the American Osteopathic Association Opportunities database to identify the aforementioned programs and those that were dual accredited in the anesthesiology, emergency medicine (EM), general surgery, internal medicine, obstetrics and gynecology, pediatrics, and psychiatry in the year 2015. Programs selected for inclusion in the study were those that were located in a rural location, self-reported a rural track, and osteopathic programs that required rural training for some or all of their graduates. The programs that remained after exclusions were then given a 55-question survey that covered the length of rural training, location, content, rural-specific content, etc. 119 programs ultimately met the criteria for inclusion, of which 82% responded to the given survey. 58% of these programs reported actively pursuing candidates with an interest in rural practice, and 56% required rural training from some or all residents. 36% (13 of 36) of programs identified as rural-centric (required at least eight weeks of rural training) were in internal medicine and 25% (nine of 25) were in general surgery. 69% of these rural-centric programs were urban-based, with 35% of them reporting only urban zip codes of their rural rotations, 54% for rural clinic sessions, and 31% for rural full-time training locations. Only two rural-centric programs were identified in obstetrics and gynecology and pediatrics, with none being found in anesthesiology. No differences in sponsoring institutions or geographic regions were noted between rural-centric and non-rural-centric programs. 53% (19 of 36) of these rural-centric programs had osteopathic accreditation. Locations were considered rural for 26% of hospital rotations and 28% of continuity clinics. Patterson et al. identified an overrepresentation of osteopathic programs among the small numbers of rural-centric programs and indicated that a successful transition to ACGME accreditation would be important to maintaining rural training in the seven specialties studied [5].

To further maintain rural training programs in various specialties, medical schools can work toward offering early exposure to rural medicine, and residency programs can further equip residents with skills to undertake the challenges of practicing rural medicine. The long-standing presence of rural-centric residency programs will not only address the physician shortage but also improve the healthcare of underserved communities. While the study itself may be somewhat hindered in its broader generalizability due to its very stringent criteria, the high response rate it received is particularly helpful. Given that this is ultimately a broad overview, it is not able to address real differences that are not readily obvious. However, what was of particular note was the way in which the definition of “rural” was not uniform across these different programs, highlighted by the fact that over half of the 36 rural-centric programs reported rural-specific training in six ABMS/ACGME core competencies [5]. Nonetheless, the information collected serves to provide a baseline for which future comparative studies can be conducted.

Medical practice and consultation in rural practice: Myhre et al. aimed to execute a comparative study of the scope of practice of family medicine graduates by practice location, rural versus urban [6]. Highlighting the shortage of rural physicians, researchers wanted to settle the perception that urban-based programs were not meeting the needs of the rural population. To evaluate the scope of practice, researchers designed a retrospective cross-sectional survey that asked questions about medical education, residency training, career history, and practice patterns after completion of their respective programs with the scope of practice being determined through domains of care (types of care, clinical procedures, practice settings, and specific populations). Participants (651) for this study were identified as graduates who had completed family medicine residency at the University of Calgary or University of Alberta from 2006 to 2011 with the actual survey being distributed from July 2013 to December 2014. These participants were recruited using the Alberta Medical Directory or the Canadian Medical Directory. The survey had an overall 47.2% (307) response from graduates, of which 173 were identified as urban program graduates and 59 as rural program graduates. The results of the survey showed that rural graduates typically had a broader scope of practice than their urban counterparts in the following practices: postnatal care, intrapartum care/deliveries, palliative care, in-hospital care, home visits, long-term care, and care for rural and indigenous populations [6]. However, irrespective of the training program location, physicians in rural practice had a broader scope of practice. This trend was further echoed in urban spaces where rural graduates were more likely to include a broader spectrum of procedures.

As past studies have shown, rural graduates are more likely to practice in rural locations comparatively. This trend is likely informed by location dynamics as researchers stated that curriculum accreditation requirements and expected outcomes for graduates of both rural and urban programs are essentially the same with differences only in training environments [6]. This suggested the possibility that these programs are likely influenced by the demands of the communities in which they reside, which is echoed by other studies in family medicine and internal medicine. Myhre et al. implied that such possibility offers rural residency programs the ability to tailor their curriculum and training to address the local needs of each rural community and their unique struggles with the physician shortage. The study itself is limited by its cross-sectional and retrospective nature, which is more susceptible to bias on the part of participants, but results speak well to the fluid and reactionary nature of training programs. Although the self-reported scope of practice was not verified, it makes for interesting speculation as to the reasons for these outcomes. Further study is warranted given these issues, but there is strong foundational information from which to draw in these future studies.

Likewise, Meyers et al. sought to quantify the rural workforce contributions of rural-trained family medicine residency graduates and compare them to the contributions of a geographically matched cohort of urban-trained graduates. They described the impact of graduate medical education (GME) on rural access to primary care physicians and used the “rural workforce year" as a means of quantifying the contributions of GME graduates to rural practice [7]. Researchers used the 2013 American Board of Family Medicine (ABFM) and American Medical Association (AMA) Physician Masterfile in order to track graduates through their practice locations. The identified sample was restricted to the years 2008 to 2018 with a self-reported specialty in family medicine. The researchers’ methodology included measurements of the total years that graduates participated in the rural workforce and the percentage of their total practice years spent in rural areas. The participants’ average rural workforce years per graduate for the rural/RTT and the matched cohort were calculated by multiplying average practice years by the average share of total workforce years in a rural location. Then, the average rural workforce years per graduate was divided by the rural rural/RYY cohort average rural workforce years per graduate to calculate how many matched graduates were needed to match the rural workforce year contribution of the rural/RTT graduates [7]. Additionally, Meyers et al. compared the average number of rural workforce years in cross sections of five, eight, and 10 years in practice after graduation. Calculations indicated that rural-trained physicians contributed a higher number of rural workforce years in total and on average per graduate compared to a matched cohort in the same practice in the same intervals. These results reiterated the amount of service provided to rural communities by RTT graduates was increased in comparison to non-rural program graduates. This emphasized the importance of residency programs in expanding rural-focused opportunities to increase the recruitment of physicians to practice in rural communities. The primary limitation of this study was its overreliance on databases, which do not make mention of any considerations that impact certain results [7]. Nonetheless, the study reaffirmed existing studies and made room for itself by quantifying the researched insights, stating that it would take nearly triple the production of non-rural graduates in order to match the contributions of one rural program graduate.

Rural Medicine in Medical Education

The necessity for physicians in rural areas is evident. Medical education is actively working toward addressing this by attempting to increase the likelihood of newly graduated physicians staying and practicing in rural communities.

Undergraduate medical education: Crump et al. sought to determine the effect of adding a rural clinical camp to their respective school in a bid to help in addressing physician maldistribution in the US. Specifically, researchers were looking at optimizing the affinity model, which proposed that when a student who is from a rural area is trained in less urbanized areas, that student is more likely to return to a small town to practice [8]. Programs following this model have typically had greater success placing graduates in rural practice. However, the appropriate amount of rural training and exposure has remained up for debate as less exposure increases the likelihood of “urban disruption.” This issue is further magnified by potential urban selections in rural-centric programs that allow student self-selection, which may come to a head with the affinity model. Rural experiences in some programs during clinical rotations vary from six weeks to as many as nine months. Researchers proposed that having rural campus options provided an additional increment in rural practice selection beyond upbringing and specialty. In order to measure this, they analyzed multiple variables (rural upbringing, demographics, campus participation and association with rural practice, and residency choice) among the graduates of both Kentucky campuses (Louisville and Madisonville) from 2001 to 2008. Analysis of the 1,120 medical school graduates reaffirmed that rural upbringing and family medicine residencies were strongly associated with rural practice. Data also indicated that rural practice only occurred with 7% of standard campus graduates, but the proportion increased to 45% of rural campus graduates [8]. Earlier reports indicated that urban disruption was more likely to occur in those who spent significant time in urban training centers. Researchers indicated in reports from the Madisonville campus that urban disruption occurred when all but six weeks of the clinical training were located in Louisville. Even though 41% of the students who attended the urban campus were from rural areas, only 9% chose rural practice [8]. The speculative nature of this study is perhaps its most significant limitation, but it manages to create a smaller framework for future studies to build upon. The analysis of only one medical school also limits generalizability, but the information it illuminates stands in line with previous research. It emphasized the role of a rural campus in influencing practice choices as seen in the top medical programs with rural tracks being based at rural clinical campuses. It is also noted that readiness to live in rural spaces was generally seen to have more influence than actual practice readiness in these decision-making processes. Researchers recommended the pooling of data from various rural-centric training programs in order to better identify subsets in data.

Observing rural medicine from a student perspective, Clark et al. aimed to determine whether rural upbringing and extended living in rural places impact a student’s intention to undertake rural practice. To understand this impact, researchers made use of the Medical Schools Outcomes Database (MSOD) and Longitudinal Tracking Project, a collaborative project headed by Deans of Australian and New Zealand Medical Schools, that follows students’ aspirations and trajectories over the course of their education and after. Part of this project included having students complete entry and exit questionnaires discussing different perspectives and goals that they had. For this study, researchers utilized data from three consecutive cohorts in the Sydney Medical Program (SMP), University of Sydney, New South Wales, making use of the questionnaires. Researchers were specifically looking at students’ initial intentions to undertake rural practice and the fulfillment of these intentions after completing their education. In total, the study utilized data from 448 students with some variation in response totals as some did not complete certain questions. The questionnaires indicated that the number of students preferring rural practice, 20.7% (79/382), dropped to 12.5% (54/433) from initial entry to completion. From these cohorts, 8.1% (35/434) had accepted rural internships despite 14.5% (60/415) indicating a preference for a rural assignment [9]. Interestingly, 98 students in total undertook extended rural placements and were more than three times as likely as students with rural backgrounds to express a preference for rural internships and more than twice as likely to accept a rural assignment. Results suggested that extended rural clinical placements had a stronger association with preference and acceptance of rural assignments than rural backgrounds, but it was noted that those students with rural backgrounds were overrepresented among those who took those extended rural placements [9]. These findings offered professionals in medical education the opportunity to serve a pivotal role in developing community-driven clinical education that places early exposure to rural medicine as its central focus. Such efforts by medical education would further increase the probability that physicians will choose to practice in rural communities following residency. Although the possible reasons for the associations are not plainly evident, the illumination nonetheless constructs a framework for more in-depth analysis. Somewhat limited by the short length of study, this creates a launching pad for a more long-term study that can produce more pertinent information. Initial assignments are typically good indicators of future career trajectories, but a long-term study provides the opportunity to venture beyond the study’s limitations. Given that the MSOD continues data collection, policy and curriculum changes will likely benefit the more that this collaboration expands.

Graduate medical education: The initial efforts of medical schools to eliminate the physician shortage in rural communities can be further addressed by medical residency programs and training opportunities. Liskowich et al. identified key components required for developing a rural family medicine training site program at two Saskatchewan test sites. In developing this framework, key academic and community stakeholders were contacted with the intention of identifying the needs for the expansion of the sites. Researchers outlined key criteria for ensuring the success of these programs, which consisted of the following: identifying a medical practice of sufficient size with exposure to subspecialty physicians, obtaining a commitment from a sufficient number of rural family physicians to act as preceptors at a rural training site, identifying the resources required by rural physician preceptors for educating medical students and residents, determining the expected additional financial and time costs to the community practices and local hospitals involved, determining if adequate outpatient and hospital facility space is available to accommodate learners, and identifying key clinical exposures for learners to assess if there is an adequate mix of hospitalized patient and outpatient exposures [10]. A mixed-methods design was used in this study, but the focus remained on the qualitative data that was collected. Participants were asked to take part in a semi-structured interview that was developed in accordance with existing literature. Those who were interviewed included community physicians, managers in Sun Country Regional Health Authority, decision-makers in the Department of Academic Family Medicine at the University of Saskatchewan (UoS), leaders in the College of Medicine at UoS, and decision-makers in the Saskatchewan Ministry of Health. Member checking was completed at the end of interviews in order to ensure the accuracy of statements that were made. It is important to note that the data analysis that was done focused more on understanding rather than casual determination, prediction, or generalization [10].

A review of the responses given in these interviews culminated in the following themes: rural placement equals rural retention; stakeholder roles and perspectives are key predictors of success; the success of a program of this magnitude requires key resources. Key resources included physical resources, physician champions (figurehead), physician teachers, educational and administration support, and specialist support. These resources were generally agreed upon and emphasized the importance of dialogue and flexibility in the overall implementation of a program. In these conversations, barriers were also discussed with differing priorities, lack of human resources, and lack of physical resources generating the most concern. Through these interviews, researchers were able to see that the long-term success of the implementation of a rural residency program is primarily contingent on the people involved rather than the physical and financial resources. While most concerns were generally agreeable among the involved stakeholders, the need for an intermediary could not be understated as opinions and end goals tended to show some deviation [10]. Given the responses from this study, there is room for rural medicine residency programs to enhance their training with the key resources mentioned to ensure physicians remain practicing in rural communities upon graduation from the residency program. The framework that is constructed struggles with generalizability due to its location-specific orientation that bends responses more toward insulated responses. The broader identified factors and resource needs that the study highlighted do provide a template for future studies at other locations. There is not anything inherently new discussed here, but it does provide a strong sense of the program installation process.

In this qualitative study, Jensen and DeWitt sought to examine the ways in which rural electives affected medical residents’ perception of rural practice and its impact on recruitment to these areas [11]. Taking into account that rural practicing physicians were more likely to come from programs in less populated areas and have partaken in a rural elective, they chose to question residents in a large university-based internal medicine practice. They elected to use residents who were completing rural electives from 1990 through 1998 (83 residents) from the University of Washington Internal Medicine Residency program, and they mailed open-ended surveys concerning the overall elective value of the aforementioned electives. Participants in rural electives were matched (gender and residency graduation year) with a group of randomly selected nonparticipants with a 70% response rate from those in electives and a 61% response rate from the nonparticipants. Survey results indicated that elective participants were comparatively more likely to have seriously considered rural practice before they began their residencies. Likert scale questions that were included in the survey revealed that breadth of practice and care continuity ranked high as encouraging factors while on-call hours and family preferences ranked the most discouraging. Participants also indicated that their interest in rural practice rose as a result, with the majority citing it as having a very prominent role in influencing their practice location preferences. In the case of those who were in urban practice, their perceptions about rural practice were often jaded by concerns of lack of access to specialists and consultants. This is where medical residency programs can implement targeted training that informs physicians in residency of ways to navigate through such challenges as limited resources and specialty care. The presence of positive role models in rural practice during electives was also highlighted as a positive influence on practice choice [11].

Overall, residents reported a positive impact from these rural electives although there was little difference in the number in rural practice between the two groups. While these findings are enlightening in some regard, the data is fairly limited in its broader application for various reasons. The researchers observed that a majority of respondents indicated that the University of Washington’s School of Medicine’s reputation for rural medicine influenced their decision to train there [11]. Further complications arise due to the study's small sample size, which causes some deviation in established findings such as the correlation of physician’s hometowns with their practice location. Still, the study serves to reaffirm the importance of rural exposure as a means of at least positively changing the perception of rural practice in programs that do not include or emphasize rural practice.

In order to understand the effectiveness of rotations in rural areas as a means of enhancing recruitment, Talley et al. conducted a quantitative study that sought to analyze the types of rural rotations available and correlate this information with rural practice after graduation. Recognizing a growing staffing shortage within these areas, this study attempted to go beyond other similar studies on the topic which tended to highlight financial incentives and look at the potential of increased rural exposure as a means of combating this worsening issue. Researchers distributed a five-question survey to EM residency program directors (126 accredited by the ACGME) over a six-month period in 2009. Participant selection purposely excluded military programs as well as newer programs that had less than two years of graduation. The survey asked directors whether rural rotations were required, elective at a predetermined location, elective with residents tasked with creating their own at non-predesignated sites, or not available. Directors were then asked how many of their graduated residents in the past two years had completed a rural rotation and were ultimately selected to work in a rural area. A total of 111 of 126 (88%) program directors provided complete answers about their programs, which accounted for 2,380 graduates over the previous two years. Results from the survey showed that only six (5%) required rural rotations, 16 (14%) offered rural electives at predetermined locations, 76 (69%) had rural rotations without predetermined locations, and 13 (12%) did not offer any possibilities for rural rotations. In the measured two-year span, 41 (37%) of these programs had at least one resident do a rural rotation, with only a total of 197 (8%), 111 of them being required to do so by their programs, completing these rural rotations. Resulting data also revealed that only 160 (7%) of graduating residents from 54 (49%) programs elected to begin their careers in rural areas, with those from programs that required rural rotations being more likely to pursue this path [12]. It is important to note that a state’s rural population was identified to be directly related to this career choice. Despite this presumed confounding factor, this study reinforced the fact that exposure to rural medicine while in training plays a major role in the recruitment of rural physicians. The information collected from this study is especially insightful in its efforts to go beyond recreating past work. The focus on identifying existing trends does well in presenting possible in-roads in which to pursue future work. Given its broad scope of participation in terms of numbers and geography, the study is fairly generalizable to its field of focus (EM) but is inhibited by its lack of direct interaction with these graduating residents. It identified potentially replicable trends that could be enacted in other programs; however, researchers noted in their discussion that the costs of implementation are potentially prohibitive and warrant further analysis.

With the purpose of providing insight into rural medicine residencies developing their training programs, Patterson et al. aimed to identify and understand the threats that rural RTT programs face to their existence and to highlight resilience factors that allow these programs to prosper. Although these programs have generally been viewed as beneficial in the long-term recruitment and retention of rural physicians, the number of RTT programs has seen some fluctuation. The number of RTTs declined from 35 to 25 from 2000 to 2010 [13]. Amid supportive efforts, that number has rebounded to 37 known operating programs as recently as 2014. Typically, these programs tend to graduate less than 100 graduates annually, which is indicative of some of the issues that these programs are forced to contend with. Despite the uptick in the total number of programs, a study of 27 RTT programs that closed between 2000 to 2004 cited difficult recruiting, leadership issues, financial factors, and other warning signs. To measure these challenges, researchers completed semi-structured interviews with RTT track leaders from 22 operational programs and two recently closed programs. The operational programs had been around for various years or were new at the time they were created; one (1980s), 10 (1900s), four (2000s), and nine (2010s). Interview questions were designed and informed by a 2014 meeting between rural medical educators. The interview questions touched upon and highlighted program strengths, resilience and sustainability, risk factors and vulnerabilities, and general advice for other RTT programs. The data collected was then analyzed in terms of pertinent and recurring themes [13].

Across assets, risks, and advice, the respondents’ answers generated the following themes (most important to the least discussed): leadership, faculty and teaching resources, program support, finances, resident recruitment, program attributes, program mission, political and environment context, and patient-related clinical experiences. It is important to note that these multiple themes tended to present together in success and failure, rather than one overriding the impact of others. While certain dangers cannot be avoided due to the nature of RTT programs such as small size and geographical isolation, these interviews emphasized the importance of the social factors and capital that are ever-present within these programs and communities. However, the most influential factor that impacted the closed programs was the loss of financial backing and sponsorship, which would be enough to sink most programs. Although the qualitative nature of this study can be limiting in some ways, it is bolstered by especially poignant responses given that those interviewed were the ones directly involved in operating and ensuring the success of these programs. Researchers suggested that the long-term viability and success of these RTT programs may rest in adjusting the ways in which they are governed (accredited, policies, etc.) [13]. This advised leadership in medical education to adapt their policies and mission to embody their efforts to address the physician shortage in rural areas of the US through various identifiable training opportunities. Given that this study works off of programs at different points in their existence, including two that no longer exist, it provides a strong body of work for the development of future RTT programs.

As a case study, Crane and Jones discussed the creation and success of the Hendersonville Family Residency Program, which was established by the Mountain Area Health Education Center (MAHEC). Created in 1994, it was a rural-track training program specifically developed with the intention of alleviating primary physician shortages in rural North Carolina areas. The program is based in North Carolina in the town of Hendersonville, which has a population of 12,000. It began small with only two residents per class and has grown to four residents per class. The program’s curriculum strongly emphasizes obstetrical and procedural training, with special attention given to practice management and community leadership. This, in turn, has led to high utilization of the teaching practice, which averages over 20,000 outpatient visits per year. High utilization and community interaction have allowed the program to better take on health leadership and service-oriented roles within the community. This community has seen it go beyond the confines of practice and make developments in service accessibility and health models. Of the 37 graduates that the program has produced since 1999, 57% practiced in North Carolina for at least three years, 65% practice in rural communities, 60% work in a location that has been identified by the U.S. Department of Health as being understaffed, and 16% are full-time faculty members of family medicine residency programs. Comparatively, the results measure significantly more success than a number of other traditional family medicine residency training programs in placing their graduates in these underserved areas [3]. The Hendersonville Family Residency Program by MAHEC serves as a prime example of how rural-focused residency programs can equip their medical residents with skills for community leadership and involvement with practice management; thus, this may yield a strong possibility for physicians from such programs to decide to practice in rural communities. While this article does not provide a significant amount of comparable quantitative data, it does provide a potential model for the creation of other programs. While this specific program does not produce an abundance of graduates due to its exceedingly small class sizes, it does nonetheless make a significant impact within its own community. It is worth considering the replication of similar small programs in rural areas to address shortages and access to service in rural areas while training residents and better acclimating them to future practice in similar spaces.

Physicians in practice following residency: Chan et al. sought to determine whether practicing rural family physicians thought they had received enough rural exposure during their residencies [14]. Given the ongoing staffing shortages affecting rural medicine practice, this study aimed to better understand changes the rural residency training programs could undertake in regard to rural exposure. Specifically, researchers measured this time in months and elected to survey practicing rural family physicians who had graduated between 1991 and 2000 from a Canadian medical school. In this study, rural was considered to include any towns with populations of up to 10,000 outside of larger urban centers. Of the 651 eligible physicians that were surveyed, only 348 (53.5%) were included after excluding those who were ineligible for varied reasons such as invalid addresses, not being in rural family medicine, not having graduated from a Canadian family medicine residency training program, etc. 33% of respondents reported graduating from rural-focused family medicine residency programs. The survey itself, which was mailed, asked questions concerning the length of rural rotations and their own opinions as to its effectiveness. Survey results indicated that participants had on average 5.3 months of rural rotations and that 58% of them felt that they had the right amount of rural exposure, which was typically six months for those in the affirmative. Results further revealed that six months was also the desired length by those who felt that they did not have enough time in rural rotations. Less than 1% of the participants indicated that they felt their rural rotations were too long. 47% of those who reported adequate rural exposure had graduated from rural-focused programs, with 13% of those indicating inadequate exposure coming from those same programs. The survey’s results reaffirmed the College of Family Physicians of Canada’s (CFPC) recommended minimum length of rural exposure, which further supported programs making this adjustment, especially for non-rural-focused programs, whose graduates tended to indicate inadequate rural exposure [14]. Although supportive of this move in all Canadian programs, especially those lacking in rural exposure, the survey itself fails to go beyond these very finite boundaries. Despite such requirements not crossing borders into U.S. medical residency programs, the concept of rural exposure could be adapted by residency programs across the country with decisions by their leadership to make it required or optional. Simply providing the option of rural rotations in the training programs by medical residencies may increase the likeliness of their graduates to practice in underserved areas, while simultaneously addressing physician shortage. Its generalizability is especially hampered by a focus on singular preferences that make tangential observations and data adaptability more difficult for future studies. This is especially true for studies that tend to focus more on skill acquisition and training.

In this comparative study, Goertzen aimed to determine whether graduates of rural family medicine residency programs felt more experienced and competent in their skills than the graduates of urban family medicine programs. Generally speaking, physicians practicing in rural areas performed more procedures than their urban counterparts. Taking into account the overall commitment to the attainment of required skills, Goertzen wanted to highlight the impact that rural residency programs had on this outcome given that surveys indicated that medical students graduated with a lack of experience and confidence in their abilities. This researcher also wanted clarification as to whether rural programs were more prone to select residents with stronger skill sets. In order to execute this study, he designed a written survey, which asked family medicine residents to report their experience and competence with 53 different procedures using five-point Likert scales. Ratings for experience ranged from “Never Observed” to “Performed Independently,” and competence ratings ranged from “Not Competent” to “Very Competent.” Participants were identified from the three urban and two rural Ontario-area family medicine residency programs. Surveys were distributed in June and July of 2000, 2001, and 2002. 77.7% (268) of residents completed the survey in 2000 and 78.1% (267) in 2001 [15]. Surveys distributed in 2002 were not included in the final data analysis due to distribution difficulties that had arisen. Response rates from graduates in rural programs (94.1%) were significantly higher than those in urban programs (73.2%). Scores drawn from the collected data were used to establish mean procedural and competence scores by which the two different resident groups were analyzed. A little less than three-quarters of the residents had completed their undergraduate studies at Ontario medical schools with similar scores between them and those who had studied elsewhere. Residents from both program types began their respective programs with an average score of 2.86 for procedural experience and an average score of 2.35 for competence. After one year of training in their programs, these scores naturally increased across the board increasing to averages of 3.36 for experience and 2.96 for competence. At the time of graduation, these scores increased further to an average of 3.77 for experience and 3.46 for competency. Upon completion of their respective programs, rural resident graduates rated higher levels of procedural experience (3.98 vs 3.70) and competence (3.67 vs 3.39) in comparison to their urban counterparts [15]. Gender was seen to be only present as a slight difference in initial scores, but rates of improvement upon graduation were comparable. However, results did reaffirm that male residents had a greater likelihood of performing more procedures than their female counterparts, which was considered to be a potential effect of professional culture or socialization. These results are especially notable given that past studies raised the speculation that rural programs recruited more experienced residents. The differences in procedural experience and competence were attributed by Goertzen to the breadth of procedural skills and knowledge demanded by rural practice, wherein specialists are not as abundant.

Furthermore, it is noted that urban teaching hospitals did not provide as many opportunities for skill acquisition as residents had to compete for them. This may be attributable to the lack of specialty care in rural communities. Hence, rural physicians adapted a broader skill set of medical practice, interventions, and procedures during their medical residency program to better care for their underserved patient population. Although the study was somewhat hampered in its generalizability by the sheer nature of self-assessment, it is nonetheless especially insightful in guiding future projects. The data set identifies specific procedures, which may identify areas of potential weakness within residency programs. The quality of the study is further bolstered by the overall response rates of the residents (94% of rural participants and 73% of urban participants) [15]. Moreover, Goertzen's study works to highlight one of the professional benefits that arise from rural practice, which may be a significant recruitment element for programs.

Scope of Practice as Rural Physicians

Though each medical specialty has its unique struggles and hurdles to overcome, the overall challenges of practicing rural medicine are shared by a majority of physicians in rural communities. This section explores the practice of family medicine, EM, and internal medicine in rural communities.

Family medicine: In this qualitative study of the factors affecting the career choices of family medicine graduates, Lu et al. sought to primarily identify the rationale for decisions affecting broader medical practice and staffing trends in Canada [16]. Of particular concern were 2004 polling results, which revealed only 24.2% of family medicine graduates were going to establish their own practices and a decline in the proportion of rural practicing physicians. In executing this study, researchers conducted two focus groups and voluntary individual interviews with graduating students enrolled in the University of Calgary’s 2004 urban and rural south streams family medicine residency program. The study was conducted from May to July 2004 with the inclusion of international medical graduates as well as the purposeful exclusion of residents enlisted with the Department of National Defense, ultimately resulting in the potential participation of 32 residents. The questions utilized in the individual interviews were developed via the two focus groups, which consisted of doctors completing rural and urban residencies. The aforementioned interviews were conducted in-person or over the phone with participation from only 18 of 32 possible participants (11 men and seven women), and the inclusion of only 17 in the final data analysis as a result of the inadvertent loss of one interview. Interviews revealed that most intended to off-put opening practices for later in their careers with all except one intending to do locums (four rural, five in a mix of urban and rural, and seven in urban only) in order to gain more experience. Reasons for partaking in rural locums primarily were geared toward gaining more experience despite intending to open practices in urban settings in the future. Of this group of 17 graduates, 15 of them intended to practice in cities, with the other two uncertain of their long-term ambitions. Social issues of family responsibilities (13/17) and lifestyle issues (12/17) primarily characterized the rationale for avoiding rural practice. Other concerns with rural practice, although not as prominent, were primarily centered on concerns with lack of readiness, finances, and the labor demands placed upon individual physicians, who expected limited professional support as a result of inaccessibility to specialists. This group of graduates only produced five individuals who intended to practice obstetrics, although the rationale for this choice was characterized by concerns with inadequate training [16].

Unfortunately, this group demonstrated a failure of the University of Calgary’s residency training program to recruit family medicine graduates to rural practice. Despite the program’s best efforts and intentions, they largely failed in their goals despite emphasizing exposure to rural medicine at both undergraduate and graduate levels. For those who participated in the urban stream, where they were not constantly based in a rural setting, the program only expected an obligatory two-month rural exposure, which was considered to potentially be too short of time to tempt future relocation to these spaces. While these results were largely reflective of past qualitative studies, the fear of inadequate training was perhaps one of the most alarming takeaways, but potentially an inroad for expanding rural exposure by providing more training in these spaces through efforts of medical education and rural medicine residency programs. The study as a whole is particularly limited in its generalizability by its small sample size, which researchers admitted was a result of bad timing on their part in the study’s execution. At this time of the year, many residents were already moving or on holiday, which was likely a major reason for lower participation. Nonetheless, the study is insightful in illuminating some of the recurring trends and factors that present themselves in similar studies. It suggests that possible emphasis on work environment and quality/type of training as potential facets to combat the more obvious mitigating factors that influence the decision of graduates to avoid rural practice [16].

In the US, Baker et al. aimed to assess the scope of practice and variations (gender, age, employment, etc.) among rural Idaho family physicians. Largely recognizing the struggles of rural physician recruitment and retention, the researchers sought to look more closely at the scope of practice, which has generally been known to be different between rural and urban physicians, with rural physicians tending to have a broader scope. Participants were asked to complete a survey, which focused on demographic questions, continuing education, scope of practice, and satisfaction. The survey was designed after a literature review and distributed via mail service. Participants for the study were identified using the contact information from the Idaho Academy of Family Physicians, Inc. (IAFP) database. Making use of this database, 275 physicians in rural counties (population of 50,000 or less) were found. 248 surveys were delivered (27 incorrect addresses) in April 2007, with the completed surveys delivered to Boise State University for processing. The survey ultimately had a 37.1% (92) response rate, with gender demographics matching the overall 2009 IAFP membership. Respondents who completed the survey provided obstetrics services in prenatal care (57.6%), vaginal delivery (52.2%), and C-sections (37%). They also indicated that they provided other operating room services (43.5%), esophagogastroduodenoscopy or colonoscopy services (22.5%), emergency room coverage (48.9%), inpatient admissions (88.9%), mental health services (90.1%), nursing home services (88%), and supervision to midlevel care providers (72.5%) [17]. Comparisons between respondents revealed that female respondents tended to be younger than male respondents, more likely to be employed, and also less likely to provide non-obstetrics related services and EGD or colonoscopy services than their male counterparts. Regression models indicated that younger physicians (ages 30-48 years old) were more likely to provide obstetrics services, be employed, have completed their training in Idaho, have loan repayment, and plan to maintain board certification in family medicine than older respondents [17]. Baker et al. highlighted the crucial role of current rural medicine residency programs in shaping the practice of physicians and their decisions to serve rural communities. Although very informational on many fronts, the study is especially hampered by its low response rate (37.1%). Even though this concern is somewhat mitigated by participation mirroring IAFP demographics, it still stands that a broader application of these perspectives may not be accurate. Moreover, while the study must contend with these limitations, it does highlight some areas for further research that may impact curriculum design for rural residency programs in the future.

Emergency medicine: Wadman et al. compared the clinical experiences of EM residents in rural emergency department (ED) rotations and those in urban university-based rotations. Rather than just affirming the benefits of rural exposure, researchers wanted to quantify this information in terms of patients evaluated, patients admitted and bed type, and type of procedures performed over six months. The urban ED selected for this study was a tertiary referral center located in a mid-western city with a population of over 400,000. With 31 beds including four trauma bays and four-room fast-track areas, this urban ED had an annual census of 42,647 [18]. The rural ED that was utilized in this study was a level II trauma center with a 12-bed facility, including three trauma bays, with an annual census of 15,283, and located in a community of 14,814 [18]. In collecting these numbers, researchers called for the participation and self-reporting of numbers by second-year EM residents completing both rural and urban rotations. Only five of six residents completed the relevant data forms that were asked of them. Rural versus urban ED patients evaluated per hour stood at 1.22 and 1.21, respectively. Rural versus urban total admission rates were 21.74% and 33.35%, telemetry admission at 3.4% and 14.24%, and ICU admission at .9% and 4.38%, respectively. Notable differences in services provided (per 100 resident-hours) between rural and urban were noted in endotracheal intubation (0 vs .9), dislocation or fracture relocation/reduction (1.3 vs .2), pediatric resuscitations (.6 vs .3), and adult trauma resuscitation (2.4 vs 3.6). The collected data showed that clinical experiences were quantitatively comparable between rural and urban EDs in regard to patients per hour and procedure frequency, but there was generally a lower patient acuity as revealed by differing admission rates [18]. The differences between rural and urban EDs may be attributable to the lack of skills and experience that rural medicine requires due to its well-known factors of limited resources and advanced care. This is where residency programs can step in to provide rural medical residents with the necessary skills, knowledge, and experience to navigate through challenging ED cases in rural areas. Although the study was well-intentioned in addressing a potential dearth in literature, it primarily only worked to reaffirm what was generally accepted knowledge. The study is further compromised by the extremely small sample size and the mere nature of self-reported results. As it were, this study went out of its way to address something that was not in dispute, but it did highlight some interesting points of embarkation in future studies.

In a short letter to the editor of The American Journal of Emergency Medicine, Cheng and Fernandez sought to highlight the disadvantages faced by rural residents in accessing adequate emergency health care. While acknowledging a general struggle in recruiting and retaining physicians in these rural spaces, they wanted to bring attention to the severity of the situation in EM in comparison to other specialties. Utilizing the 2001-2002 Graduate Medical Education Database, Cheng and Fernandez executed a retrospective descriptive comparison of 10 different residency specialties in the 12 most rural states according to population density (Alaska, Idaho, Kansas, Montana, Nebraska, Nevada, New Mexico, North Dakota, South Dakota, Oregon, Utah, and Wyoming). The 10 different specialties (EM, anesthesiology, family practice, internal medicine, neurosurgery, orthopedics, pediatrics, psychiatry, surgery, and obstetrics & gynecology) were divided into three categories: number of rural states represented by each specialty, percentages of respective residency programs found in these rural states for each specialty, and the percentage of residents found in these rural states for each specialty. The resulting data demonstrated that only two of the 12 rural states had an EM residency program with the percentages of these programs in the aforementioned states being 1.68%. Residents in these EM programs represented 1.23% of the total residents in these rural states [19]. The lack of representation in this field likely stems from a lack of available programs. In 1970, there was only one EM residency and one state represented; however, in 2003, there were 127 EM residencies with 35 states represented [19]. Cheng and Fernandez further hypothesized that the inability in these states to recruit board-certified emergency physicians may be part of the reason for this lack of development within this field. From the efforts of Cheng and Fernandez, it was most evident that rural-focused targeted training in current residency programs is needed to address the lack of available programs in rural areas. The rural-focused targeted training may serve as just an aspect of the overall established residency program; however, it will enable and influence more physicians to practice in rural communities across the US. It is also potentially influenced by a lack of hospitals meeting the Residency Reviews Committee’s EM department visit criteria. While these are potential reasons for the lack of EM residency program development, the various factors impacting this situation are not readily identified in this correspondence. It serves to spotlight and reaffirm the lack of accessibility to these specialists but does not go any further. There is no solution to be offered here, but nonetheless, this proves potentially beneficial in exposing more spaces for improvement in terms of residency training program development.

Internal medicine: Dick III et al. sought to identify factors in internal medicine residency training that impact career choice and determine the extent of their influence. Taking into regard past studies that focused on recruitment factors, researchers noted that few studies focused on ways to maintain residents’ interest in primary care during their residencies. In order to identify which factors affected career choice during residency, researchers conducted a retrospective cohort study at a large internal medicine residency program and reviewed self-reported career plans upon graduation. Residency files with the desired information on all graduates from the year 1996 to 2006 were reviewed for the timing of ambulatory clinic rotations during the intern year and 2nd year and a rural primary care rotation during 2nd or 3rd year, in addition to gender, residency track (categorical or primary care), and career plan (primary care or other) at end of residency [20]. The independent variables that were identified included curricular data (categorical or primary care), timing of clinic block, rural training experience, gender, and year of graduation. In total, 451 residents completed all internal medicine training in the review time frame, with 53% having completed the categorical track and the other 47% accounting for primary care tracks. Of the categorical track graduates (237), 22% planned to pursue primary care, and 51% of the primary care graduates planned to do the same. 39% of the categorical track graduates completed a rural training rotation and 35% of the primary care track residents completed a rural rotation [20]. Further analysis of the resulting data revealed that residents who completed a rural rotation were twice as likely to follow a career path in primary care in comparison to those who did not. Researchers suggested that this likely results from factors such as more active exposure to the breadth of practice and stronger relationships formed with mentors, but they are quick to point out that their study did not gauge initial interests prior to beginning the aforementioned rotations or opinion changes after. It was also noted that earlier clinic rotation was not seen to be strongly associated with an increased interest in primary care [20].

Of particular note is the fact that this group of residents reaffirmed a rapid national trend of decreasing interest in primary care career paths, which is especially notable given the notoriety of the primary care in the program. Although the study is slightly limited in its generalizability due to its focus on a single program, the large number of graduates reduced the impact of this limitation. However, data on prospective careers in primary care are reflective of previous studies, which show a growing problem in this area. This is further bolstered by observations of career changes into specialization only a few years after primary care practice. Researchers believe that potential remedies to this issue are increasing the number of primary care residents and increasing the number of rural training rotations. Given its concerted efforts to analyze and compare its observations with national trends and past studies, this study is especially useful in informing future decisions about the topic. The self-awareness of the researchers greatly improves the contextualization of the data that it has collected [20].

Addressing Physician Storage in Rural Communities

The physician crisis in rural communities of the US is starkly evident. The limited healthcare access is compounded by challenges in recruiting physicians to practice in such areas, specialty care located many miles away, and dwindling resources. Numerous factors have led to the physician shortage present in rural communities; however, there are opportunities to solve this problem through enhancing rural residency programs and targeted physician training.

Factors leading to physician shortage: Helland et al. sought to examine and identify various factors that impact EM residency graduate decisions to practice in rural areas with a survey study. The decision to focus exclusively on residency-trained EM physicians was borne of past studies, which primarily focused on family physicians. The study was further warranted given recent studies that identified a shortage of trained emergency physicians and accessibility issues for patients in rural settings compared to those in urban areas. Participants of this study were selected via convenience sampling utilizing the AMA Physician Masterfile, which identified EM residency graduates and provided contact information for them. From this pool of potential participants, those who did not actively practice EM in clinical practice or were military personnel (limited practice location choices) were purposely excluded. Further exclusions were enacted if selected graduates had moved or had military commitments. The rural sample consisted of EM residency graduates from 2006 to 2008 and the urban sample was selected at random. After enacting the aforementioned exclusions, the initial sample of 164 urban and 164 rural EM residency graduates was reduced to 296 participants (145 urban and 151 rural) [21]. Definitions of rural and urban were established using the United States Department of Agriculture (USDA) county-based Urban Influence Codes. The survey distributed to these participants was developed in accordance with past studies, although the questions included in the survey were not validated with past data despite being piloted and refined through feedback from residents and faculty not eligible for participation. The survey asked participants to rate the importance of 13 factors that potentially affected their practice location choices and the overall influence they had on the selection process. The 13 factors considered by the survey were previous time spent in a similar area, family/spouse, cost of living, lifestyle, access to amenities/recreation, rotation/experience during residency, salary/signing bonus, loan repayment, access to continuing medical education, service to the underserved, autonomy/scope of practice, access to specialists, and ED volume/acuity. Survey results showed that lifestyle (98%), access to amenities/recreation (95%), ED volume/acuity (93%), and family/spouse (90%) were highlighted as somewhat or very important in graduates’ selection processes. Access to specialists was identified as the biggest difference between urban (very important at 44%) and rural groups (very important at 20%). Researchers also found that more rural physicians spent their entire childhood in rural spaces in comparison to those in urban settings [21].

Ultimately, this survey worked to demonstrate that urban emergency physicians were more likely to consider rural practice relative to familial connections, monetary incentives, and increased access to specialists. Of those who did not participate in a rural rotation during their residency, 44% would have had the option available [21]. The researchers identified and studied 13 key factors that continue to impact and hinder the decision of graduated residents to practice in rural or urban areas. With more targeted efforts, rural medicine residency programs can address these 13 factors by equipping residents with resources and skills to strengthen recruitment to rural communities and address the physician shortage. While all of this information is insightful, the study is limited by its structural and social limitations. Given that the survey only had a 67% response rate, its larger application is hindered by potential response bias. However, the larger issue with this study comes from inconsistencies on the part of participants who had inconsistent definitions of rural and urban. This likely arose from the fact that participants hailed from 47 different states [21]. Although the survey used USDA definitions, the interpretation on the part of participants warps the consistency of the results. This becomes especially problematic in broader generalizability that is already hampered by the major role of responses to hypothetical situations that could potentially see deviations from reported answers. Nonetheless, this study worked well in providing insights into a niche that can often be neglected and underdeveloped in studies.

Potential solutions to physician shortage: In order to provide medical education and an understanding of key elements to increase the proportion of physicians who will choose to practice in rural communities, Morken et al. sought to identify the factors that influence physician recruitment and retention upon completion of RTT residency programs. Researchers noted that a number of these programs have come about as a means of addressing the ongoing shortage of physicians in rural areas. Acknowledging the success that these programs have had in improving recruitment and retention issues with over 70% of graduates being placed in rural areas, they note that the reasons for these improvements are not evidently clear [22]. The importance of identifying these factors is further emphasized by noting that ongoing changes to the healthcare system warrant discussion as to whether decision-influencing factors are changing as well. Researchers conducted this case study with the University of Wisconsin-Baraboo Rural Training Track Family Medicine Residency Program due to the program’s high recruitment rates to rural spaces. A cross-section survey was employed to identify factors that this group of graduates found important in their retention in these areas. 16 questions highlighting physicians’ views about these factors as well as demographic information made up the resulting data. The factors that the study was focusing on were drawn from past studies. Of the 26 physicians who were invited to participate, only 19 responded, and three of them were excluded due to them only practicing in urban areas. Of the physicians that had ever practiced in rural areas, 87.5% of them were still practicing in these areas and others like them, with half of them still working at their original practice sites [22]. None of the participants made the move from urban to rural, with most past and future movements being characterized by rural-to-rural relocations. Of note is that responses to some demographic questions indicated that nine (56.3%) were female, nine (56.3%) grew up in rural communities, and 12 (75%) wanted to become rural physicians prior to beginning their residency program. The retention rates presented by the study are consistent with those of other RTT program graduates. The top 5 factors identified as very important or extremely important on average were 1) significant other’s wishes, 2) meaningful work, 3) local community, 4) medical community/work environment, and 5) work/life balance [22]. The themes mentioned in the study were consistent with past research, but there was some variation in that teaching opportunities and loan repayment opportunities were ranked as the least important, which caused researchers to surmise that financial opportunities are more closely tied to recruitment rather than retention. Although this study presents interesting insights, it is unfortunately, particularly limited in its generalizability due to its small sample size. The small size provides a better representation of regional perspectives rather than larger national rationales. However, the fact that a majority of those who graduated from this RTT intended to pursue this path beforehand might shine a better light on the ideal candidates to pursue other programs. Given that RTTs are likely to already draw these types of candidates, it would be worthwhile for residency programs to spend more time identifying other shared characteristics that may help develop a profile for the ideal candidate who is more likely to establish themselves within rural communities.

Rourke et al. sought to collect opinions from family medicine residents and practicing rural physicians on potential solutions to address a shortage of healthcare providers in rural areas. While researchers make assumptions as to what may be causing these issues with recruitment and retention, they primarily focus on the potential solutions to relieve the problem. The study itself consisted of pilot-tested questionnaires, which were mailed out in November 1999 to a large group of 507 practicing rural physicians and 536 first- and second-year residents. The questionnaires asked participants to rate the importance of the suggested solutions they proposed as well as to rate return-of-service agreements and medical education. In identifying and selecting potential participants, researchers used the Ontario Medical Association (OMA) definition of rural practice as those working “in communities with a population of less than 10,000 more than 80km away from a community of at least 50,000” [23]. Suggested solutions for changes and investments in medical education that were presented to participants included the implementation of undergraduate return-of-service agreements, postgraduate rural medical training, training rural teachers, advanced skills training, reentry training positions, and continuing medical education. It is important to note that a few questions were restricted to a specific group. Suggestions for rural practice solutions included changes in approaches to referral and support networks, medical informatics, physician licensure, allied health professionals, clinical support programs, remuneration, limitations and burnout, spousal and family concerns, and locum programs. The study saw an overall response rate of 46.6% (276 of 507 physicians and 210 of 536 family medicine residents). The solutions were ranked utilizing mean rating scores with all solutions rated as “very important” by 50% or more of at least one group. This criterion was met by all education solutions and all but two of the practice solutions [23]. Significant differences were only identified with four of the 31 total solutions, but of particular note is the fact that only 29 of the 210 who responded indicated a willingness to practice in rural areas. Of the 31 total solutions, continued funding for OMA learner-driven continued medical education (CME) for rural physicians and an alternate payment plan to include allotted time off to attend and teach CME for residents were rated as the most popular selections [23]. Other prominent mentions included limiting on-call work to no more than one night in five and a provincial locum. While this study is insightful in echoing the results of past studies on the subject matter, its best attribute is its approach in attempting to evaluate solutions rather than just reaffirming what are becoming known problems. This provides leadership of medical education and rural medicine residency programs to implement such solutions and adapt them to their unique efforts in improving the healthcare of underserved communities. However, given that the effectiveness of the proposed solutions was not evaluated, the study cannot truly be generalized beyond providing potential solutions for others to replicate. These concerns of generalizability are further bolstered by a smaller level of participation than what was originally sought. Nonetheless, this study could potentially provide opportunities for expanding ongoing work and the reception to possible recommended changes.

Discussion

Practical Implications

This review emphasized the pivotal role of medical schools and residency programs in addressing the gaps in rural medicine. The current research portrays the role of medical education and rural medicine residency programs in addressing the physician shortage and offering more support to current rural physicians. As evidenced by the studies mentioned in this review, there is a need for sustainable interventions that support physicians practicing in rural resource-limited areas while increasing the physician workforce in those areas. Practical solutions recommended for improving rural medical practice include early exposure to rural medicine during medical school, adequate mentorship during residency, increased access to specialists for referrals, monetary benefits, and healthy work-life balance [4,8,11,12,14,15,18,21-23].

Limitations

Several limitations existed in this review. A primary limitation was that many studies lacked a robust sample size, which limited their generalizability to rural communities on a national level [15-17,21-23]. These studies were conducted in various rural parts of the US, each with its own unique hardships in terms of resource availability, patient demands, and social norms, which may have played a role in the struggles of practicing rural physicians.

Future Research

Though the current literature provides insight into the impact of rural medicine residencies on addressing the physician shortage, there are several opportunities for prospective studies to investigate this impact. An in-depth analysis of physicians’ perspectives from the start of residency to the transition into practicing in a rural area can serve to thoroughly understand the healthcare challenges and needs of rural communities in terms of patient care. Also, addressing and reviewing the appropriate medical responses or lack thereof could be beneficial when viewing the tools and accessibility to healthcare in rural versus urban settings. Future research should aim to address the gaps in research on the healthcare stance in rural communities and how to further support rural practicing physicians.

## Conclusions

Rural areas of the US have limited access to health care. As evidenced by this review, numerous factors contribute to this predicament. This review sought to understand the current situation of rural medicine and physician shortage in rural areas from a medical education perspective. Effective measures to alleviate the current physician shortage prioritized the essential role of rural-focused medical education and residency programs. By equipping medical residents with the skills and experience to navigate the unique challenges of rural healthcare, such programs increase the likelihood that physicians will choose to practice in these communities. Expanding rural-focused medical education and residency opportunities can strengthen recruitment efforts and ultimately improve healthcare access for rural populations. These initiatives are vital to addressing the physician crisis in rural Kansas and other similar regions across the US. These studies offered insight into effective measures that can be employed in addressing the current physician shortage in rural Kansas. It is imperative to underscore the continued need for collaborative efforts from medical schools, residency programs, and employers to address underserved communities in rural areas of the US.
